# Evaluating the Use of Mindfulness and Yoga Training on Forensic Inpatients: A Pilot Study

**DOI:** 10.3389/fpsyt.2020.614409

**Published:** 2020-12-10

**Authors:** Christina Spinelli, Etienne Paradis-Gagné, Megan Per, Matthew H. Fleischmann, Viktoriya Manova, Aimée Wallace, Bassam Khoury

**Affiliations:** ^1^Department of Educational and Counselling Psychology, McGill University, Montreal, QC, Canada; ^2^Faculty of Nursing, Université de Montréal, Montreal, QC, Canada

**Keywords:** mindfulness, yoga, forensic inpatients, stress, cognitive and emotion regulation

## Abstract

Forensic inpatients (i. e., individuals found not responsible for a criminal offense on account of mental illness) represent an often marginalized and difficult-to-treat population. This has led to the need for research exploring the effectiveness of novel interventions. A Canadian forensic hospital has developed an 8-weeks mindfulness and yoga training program (MTP). This pilot study examined the potential effects of this program on patients' mindfulness, stress, and use of cognitive and emotion regulation strategies. A sample of 13 forensic inpatients (male = 92%) participating in the MTP program completed self-report measures assessing dispositional mindfulness, perceived stress, and use of cognitive emotion regulation strategies at baseline, post-intervention, and a 3-months follow-up. Repeated measure ANOVAs found a significant increase in the describe facet of mindfulness (*p* = 0.03) with a large effect size (η_p_^2^ = 0.26) and a significant decrease in stress (*p* = 0.003) with a large effect size (η_p_^2^ = 0.39). Pairwise comparisons revealed medium to large significant changes between baseline and post-intervention for both the describe facet (*p* = 0.03, Hedge's *g* = 0.55) and stress (*p* = 0.003, Hedge's *g* = 0.70). However, comparisons were insignificant between baseline and follow-up. No significant main effects were found on the use of cognitive emotion regulation strategies. This pilot study offers preliminary support for the use of the MTP as an adjunctive therapy in forensic inpatient treatment. Further investigation is needed into the long-term impacts of this training.

## Introduction

Mindfulness is the process of being purposefully present, alert, and attentive in the moment (1). The goal of mindfulness is to teach one to self-observe in an objective and detached manner and to be reflective rather than reflexive (2–4). One modality of practicing mindfulness is yoga, which has been described as mindfulness in motion and encompasses a group of integrative physical and spiritual practices with the goal to transform the mind and body (2, 5). Unlike many other mindfulness strategies which tend to be sedentary, yoga involves movement and focuses awareness onto one's physical body, and one's motion in the present. Scientific reports on mindfulness training support its use as an intervention for a variety of physical and psychological difficulties (6–8). In particular, yoga is suggested to be an effective treatment for stress, anxiety, depression, and substance abuse (2, 5, 9–11).

Despite the growing interest in mindfulness research on various settings and populations, the research on the effectiveness of mindfulness-based and yoga interventions for forensic inpatient populations is in its infancy. These individuals have been found not criminally responsible for an offense on account of mental illness and are sent to a forensic facility for treatment (12). As they represent a marginalized population and can be considered difficult-to-treat, there is a need to explore adjunctive treatments (13). Mindfulness has been argued to be effective as it reduces risk factors and addresses three main concerns: (1) poor affective self-regulation, (2) poor anger control, and (3) impulsivity (14).

Furthermore, forensic inpatients share similarities with incarcerated and psychiatric populations. First, forensic inpatients have committed a criminal offense and are mandated to be in a psychiatric facility. Second, the National Trajectory Project examined the NCR population in Canada and found (1) the most common primary diagnosis was a psychotic spectrum disorder, (2) 33% had a severe mental illness and an associated substance use disorder, and (3) 72% had at least one prior psychiatric hospitalization (15). Mindfulness interventions have been used with both incarcerated [e.g., (16–22)] and psychiatric [e.g., (23–27)] populations, and there is a larger body of research into their effectiveness.

In incarcerated populations, it is proposed that mindfulness training offers a healthy coping mechanism for inmates to better manage the stress of prison life and be less reactive to intense emotional states. A recent meta-analysis found that mindfulness-based interventions significantly reduce psychological outcomes (e.g., depression) and criminogenic needs (e.g., impulsivity) in pre-post studies (28). A pilot RCT also found reductions in risk behavior following release from incarceration (29). Small but positive effects of yoga on psychological behavioral functioning have also been found (2). For populations with severe mental illness (including psychosis), it has been proposed that mindfulness aids in alleviating the distress associated with psychosis by helping patients relate differently to their psychotic experience (8, 30). Individuals learn to see psychotic sensations as transient and recognize these sensations do not fundamentally define them as individuals (31). Khoury et al. (8) found a moderate effect of mindfulness interventions on reducing negative symptoms of psychosis and increasing functioning and quality of life. Mindfulness practice also appears feasible and acceptable for inpatients, with more active interventions (e.g., yoga) possibly being most appropriate (26).

Taken together, mindfulness and yoga training appear beneficial for incarcerated populations and individuals with severe mental illness. This adds to the argument that such training can be useful adjunctive interventions with forensic inpatients. However, the present literature is limited. Singh et al. (32) examined the effects of a mindfulness meditation program on aggression in forensic in patients with mild intellectual disabilities. Data collected by staff members indicated substantial reductions in physical and verbal aggression. A more recent mixed-methods study by Singh et al. (33) examined mindfulness meditation as treatment for adult sexual offenders with intellectual disabilities in a forensic facility. They found that the three participants were more successful at controlling their deviant sexual arousal when using the learned mindfulness exercises.

Most recently, Sistig et al. (34) conducted a preliminary evaluation of mindful-yoga as an adjunctive treatment for forensic inpatients in New Zealand. To our knowledge, this study is the first to assess the use of mindful-yoga with forensic inpatient adults. They examined the effectiveness of an 8-weeks mindful-yoga program on 26 inpatients and conducted baseline, post-intervention, and 2-months follow-up assessments. A mixed-methods design was used to examine trait mindfulness, stress, anxiety, depression, and general distress. 92% of participants reported that the delivery of the program was acceptable. In addition, they found subjective increases in body and breath awareness, greater relaxation, and a trend toward reduced anxiety and increased mindfulness (on the *observing* facet). Although the study lacked statistically significant results, benefit-consistent trends were found. Furthermore, the qualitative findings suggest the suitability of mindful-yoga for forensic patients and that there are perceived physical, emotional, and social benefits of this program.

The research on mindfulness and yoga as adjunctive therapies for forensic inpatients is limited both in number and by methodological concerns; indicating a need for further research. There is also a lack of research examining Canadian populations. Currently, a Canadian forensic hospital runs an 8-weeks mindfulness and yoga training program (MTP) for adults suffering from various psychiatric disorders (e.g., schizophrenia, personality disorders). We conducted a pilot study examining the program's effects on patients' mindfulness, stress, and cognitive and emotional regulation strategies.

## Materials and Methods

### Participants

Thirty-six adult forensic inpatients expressed interest in participation. They were included in the study if they had self-selected to be enrolled in an 8-weeks mindfulness training program (MTP) and were 18 years or older. In addition, the hospital staff could exclude any individual when clinical judgment deemed them unsuitable to participate. Twenty-three were removed because they either did not complete the consent form (*n* = 3), did not complete the measures at all timepoints (*n* = 15), completed the MTP previously (*n* = 2), attended <3 sessions (*n* = 2), or found out they were leaving the facility and stated this likely impacted their results (*n* = 1). This left 13 participants in the final analysis.

### Intervention

The study took place at a Canadian forensic hospital, and the MTP was developed by the staff of the hospital with the objectives of enhancing the practice of meditation and mindfulness, establishing a sense of calm and serenity, developing concentration and creativity, and learning to be aware of and manage anxiety as well as other negative emotions. Twice a year, two MTP groups were facilitated concurrently; leading to four periods of recruitment across 2 years (2016 to 2018). The program was designed as an adjunctive therapy and all participants continued to receive their psychiatric treatment-as-usual. Each session was 1.5–2 h long and included meditation and/or yoga practice. A brief description of the 8-weeks syllabus can be found in Table 1. During the first session, participants received a CD containing mindfulness and yoga exercises they could practice outside the sessions. Participants also received ongoing support and tools as well as readings throughout each session. This program had been established for 4 years prior to the start of this study and was facilitated by the two developers: (1) a nurse-clinician trained in mindfulness therapeutic practices, and (2) a specialized educator who is also a yoga instructor. Both were employed at the hospital and had worked in mental health for over 25 years.

**Table 1:** Description of the weekly activities in the mindfulness training program.

Session 1:	Introduction to mindfulness concepts and its use in stress management. Practice with seated and walking meditation, and relaxation.
Session 2:	Development of breath and body awareness through guided meditation. Exploration of how the mind and body can influence health and healing.
Session 3:	Building consciousness of interbeing and interdependence (i.e., how everything around us interacts together).
Session 4:	Development of awareness toward sensations (i.e., hearing, sight, smell, taste, touch) through guided exercises.
Session 5:	Development of awareness toward emotions and management of suffering. Exploration of how perception can create suffering and what to do when others are suffering.
Session 6:	Building anger management skills. Exploration of sources of anger, how to accept and manage it, and how to be in the presence of someone who may be experiencing anger.
Session 7:	Development of awareness toward thoughts and how to manage them. Discussion of concepts like “thoughts are not facts.”
Session 8:	Summarizing activities and concepts from previous weeks. Evaluation and feedback from participants.

*Each week included guided meditation and/or yoga practice*.

### Procedure

The MTP was presented by the two facilitators on all units of the hospital. Those who were interested could then subscribe to the program. Patients who could not leave their units for clinical reasons (e.g., high risk of violence or psychotic decompensation) were not able to participate in the program as it was held in a room outside of the units. The facilitators had discussions with the care team and the unit psychiatrist to identify participants who would be eligible to participate.

Participants enrolled in the MTP were then approached by the clinic nurse specialist (E.P.G.) and a trained research assistant. Patients were verbally informed of the study and were explicitly told that their decision to accept or decline to participate would not impact their spot in the MTP. Those agreeing to participate gave informed consent and completed measures in English or French at baseline, post-MTP, and 3-months follow-up. Participants were assisted when filling in the questionnaires and were able to take breaks. We initially sought to obtain a convenience sample of control participants by asking individuals who were not interested in participating in the MTP or were not enrolled at that time if they would be interested in completing questionnaires. However, this was not possible due to low interest. All procedures were approved by the Research Ethics Board at the hospital and McGill University.

### Measures

#### Demographics

A brief questionnaire with questions about age, diagnosis, race, sex, and hospitalizations. Information was obtained through self-report and consulting medical records.

#### Mindfulness

The Five Facet Mindfulness Questionnaire [FFMQ; (35, 36)] is a 39-item measure of dispositional mindfulness that assesses five facets, specifically: (1) observing, (2) describing, (3) acting with awareness, (4) non-judging of, and (5) non-reactivity to inner experience. A 5-point Likert scale is used and ranges from *Never or Very Rarely True* to *Very often or Always True*. The FFMQ has been previously used with inpatient forensic populations (34) and other clinical populations (37). The psychometric properties of this measure were generally good across timepoints [*total score* [α = 0.86 to α = 0.94], *describing* [α = 0.81 to α = 0.92], *acting with awareness* [α = 0.84 to α = 0.89], and *non-judging* [α = 0.79 to α = 0.83]]. However, the *observing* (α = 0.52 to α = 0.83) and *non-reactivity* facets (α = 0.49 to α = 0.80) had poorer psychometric properties; suggesting the need for caution during interpretation.

#### Stress

The Perceived Stress Scale [PSS; (38–40)] is a 10-item measure that evaluates the extent one sees common life situations as being stressful over the last month. A 5-point Likert scale is used and ranges from *Never* to *Very Often*. The PSS has been used with forensic (34) and incarcerated populations (17). There was strong internal consistency (ranging from α = 0.81 to α = 0.93) within this sample across the three timepoints.

#### Cognitive and Emotional Regulation

The Cognitive Emotion Regulation Questionnaire [CERQ; (41, 42)] is a 36-item questionnaire that evaluates nine cognitive and emotion regulation strategies used when responding to negative circumstances. A 5-point Likert scale is used and ranges from *Almost Never* to *Almost Always*. Strong properties were found with *positive refocusing* (α = 0.90 to α = 0.95), *positive reappraisal* (α = 0.79 to α = 0.90), and *other-blame* (α = 0.81 to α = 0.89). The psychometric strength varied from acceptable to strong depending on the time point for *acceptance* (α = 0.59 to α = 0.74), *refocus on planning* (α = 0.63 to α = 0.95), *putting into perspective* (α = 0.52 to α = 0.86)], *catastrophizing* (α = 0.64 to α = 0.86), and *rumination* (α = 0.50 to α = 0.82). Finally, *self-blame*'s psychometric properties were inadequate (α < 0.5); as such, this subscale was removed from our analyses.

### Data Analysis

One-way repeated-measures analysis of variances (ANOVA) were conducted using IBM SPSS Statistics (Version 25 for Windows). Pairwise comparisons were used to contrast timepoints. In addition, Bayesian repeated measure ANOVAs were conducted using JASP (Version 0.14 for Windows) to further assess our findings (43). Uninformative prior odds were used due to the scarcity and limitations of previous studies. One missing value was resolved through subscale mean replacement. An outlier (±3.5 SD) was removed from the *observing* and *non-reactivity* facets.

## Results

### Demographics

The sample was predominately male (92%), ranging in age from 19 to 48 years (*M*_*age*_ = 33.54, *SD* = 9.87). The majority of the participants were white (*n* = 10, 76.9%) and three participants (23.1%) were from another racial background. The average number of years spent in school were 10.5 (*SD* = 2.71; Range = 8–17 years). The majority of participants had a primary psychiatric diagnosis of schizophrenia (*n* = 5, 38.5%), followed by schizoaffective disorder (*n* = 3, 23%). A substantial number of participants (*n* = 8, 61.5%) had more than one psychiatric diagnosis and 38.5% of participants (*n* = 5) had comorbidity with a personality disorder and/or substance-use disorder. Clozapine (*n* = 4, 31%) was the most frequently prescribed antipsychotic medication. Most participants committed a form of assault (*n* = 9; 69.2%) followed by murder (*n* = 3, 23.1%). 38.5% of participants had no prior history of arrest (*n* = 5), 30.8% of participants reported one prior arrest (*n* = 4), and 23.1% of participants reported two prior arrest incidents (*n* = 2). The average number of prior hospitalizations were 5.27 and ranged from 1 to 20 visits. Most participants were enrolled in other treatment programs in the facility such as individual psychotherapy (*n* = 7; 53.8%), relapse prevention program (*n* = 4; 30.8%), and anger management program (*n* = 2; 15.4%). Individual psychotherapy is offered by a team of psychologists using different psychotherapy approaches. The relapse prevention and anger management groups are facilitated by clinicians and utilize motivational and cognitive-behavioral approaches, respectively.

### Intervention Effects on Measured Outcomes

Mean values, standard deviations, and effect sizes (partial eta squared effect sizes and Hedge's *g*) for all outcomes (i.e., mindfulness facets, stress, and cognitive emotion regulation strategies) at each time point as well as the one-way repeated measures ANOVA statistics can be found in Table 2. Additional pairwise comparisons can be found in Table 3.

**Table 2:** Statistics of one-way repeated-measures ANOVA tests for the total score on mindfulness (and its five facets), perceived stress, and cognitive emotion regulation strategies.

**Measure**	**Baseline**	**Post-intervention**	**Follow-up**	**ANOVA**		
**FFMQ**	***M (SD)***	***M (SD)***	***M (SD)***	***F*_**(2, 24)**_**	***p***	**η_p_^2^**
Total Score	133.73 (15.80)	140.46 (16.25)	140.00 (19.90)	1.23	0.31	0.09
Acting with awareness	28.77 (5.57)	30.31 (6.03)	30.15 (5.10)	0.75	0.49	0.06
Describing	25.31 (4.97)	28.38 (5.38)	28.08 (5.30)	4.25	0.03^*^	0.26
Non-judging	27.54 (5.97)	26.62 (5.95)	28.04 (4.89)	0.43	0.66	0.03
	***M (SD)***	***M (SD)***	***M (SD)***	***F***_**(2, 22)**_	***p***	**η_p_^2^**
Non-reactivity	23.71 (4.52)	26.08 (2.75)	24.63 (4.42)	1.21	0.32	0.10
Observing	29.33 (4.05)	31.33 (2.64)	30.67 (4.31)	1.05	0.37	0.09
**CERQ**	***M (SD)***	***M (SD)***	***M (SD)***	***F***_**(2, 24)**_	***p***	**η_p_^2^**
Catastrophizing	8.73 (4.01)	9.69 (2.93)	10.18 (3.13)	1.14	0.34	0.09
Other-blame	8.54 (3.43)	8.15 (2.97)	8.23 (2.40)	0.09	0.91	0.01
Positive re-appraisal	14.77 (3.39)	15.62 (3.78)	14.77 (4.49)	0.51	0.61	0.04
Positive refocusing	11.08 (4.05)	11.77 (4.53)	13.65 (4.21)	2.37	0.12	0.17
Putting into perspective	11.04 (4.07)	12.77 (2.80)	13.12 (4.02)	1.85	0.18	0.13
Refocus on planning	13.85 (2.85)	15.54 (3.23)	14.5 (3.71)	1.52	0.24	0.11
Rumination	11.23 (3.54)	11.62 (2.96)	11.54 (2.67)	0.09	0.92	0.01
	***M (SD)***	***M (SD)***	***M (SD)***	***F***_**(1.4, 16.8)**_	***p***	**η_p_^2^**
Acceptance	14.08 (3.25)	13.77 (3.17)	13.46 (3.02)	0.19	0.75	0.02
**PSS**	***M (SD)***	***M (SD)***	***M (SD)***	***F***_**(2, 24)**_	***p***	**η_p_^2^**
Perceived Stress	15.23 (5.84)	10.69 (6.26)	13.00 (6.51)	7.75	0.003^*^	0.39

* *p < 0.05. CERQ, Cognitive Emotion Regulation Questionnaire; FFMQ, Five-facet Mindfulness Questionnaire; PSS, Perceived Stress Scale. A small effect size (η_p_^2^) is 0.01, medium is 0.06, and a large is 0.14*.

*Means, SDs, F and p-values, and partial eta squared effect sizes (η_p_^2^) are listed*.

**Table 3:** Pairwise comparisons at baseline, post-intervention and follow-up for the total score on mindfulness (and its five facets, perceived stress, and cognitive emotion regulation strategies.

**Measure**	**Baseline to post-intervention**			**Baseline to follow-up**		
**FFMQ**	**Mean difference**	***p***	***g***	**Mean difference**	***p***	***g***
Total score	6.73	0.40	0.39	6.27	0.93	0.32
Acting with awareness	1.54	0.93	0.25	1.39	1.00	0.24
Describing	3.08^*^	0.03	0.55	2.77	0.12	0.50
Non-judging	−0.92	1.00	−0.14	0.50	1.00	0.08
Non-reactivity	2.38	0.31	0.52	0.92	1.00	0.19
Observing	2.00	0.45	0.50	1.33	1.00	0.30
**CERQ**	**Mean difference**	***p***	***g***	**Mean difference**	***p***	***g***
Acceptance	−0.31	1.00	−0.09	−0.62	1.00	−0.18
Catastrophizing	0.96	0.97	−0.24	1.45	0.68	−0.36
Other–blame	−0.38	1.00	0.11	−0.31	1.00	0.09
Positive re-appraisal	0.85	1.00	0.22	0.00	1.00	0.00
Positive refocusing	0.69	1.00	0.15	2.58	0.24	0.58
Putting into perspective	1.73	0.67	0.43	2.1	0.20	0.48
Refocus on planning	1.69	0.33	0.51	0.65	1.00	0.18
Rumination	0.38	1.00	−0.11	0.31	1.00	−0.09
**PSS**	**Mean difference**	***p***	***g***	**Mean difference**	***p***	***g***
Perceived stress	−4.54^*^	0.003	0.70	−2.23	0.35	0.34

* *p < 0.05. CERQ, Cognitive Emotion Regulation Questionnaire; FFMQ, Five-facet Mindfulness Questionnaire; PSS, Perceived Stress Scale. A small effect size (Hedge's g) is 0.20, medium is 0.50, and large is 0.80*.

*Mean difference, p-values, and Hedge's g effect sizes are listed*.

A large significant main effect of the MTP was found on the *describing* facet, *F*_(2, 24)_ = 4.25, *p* = 0.03, η_p_^2^ = 0.26 and on stress, *F*_(2, 24)_ = 7.75, *p* = 0.003, η_p_^2^ = 0.39. Further pairwise comparisons (using a Bonferroni correction) revealed a significant increase between baseline and post-intervention for *describing* (*p* = 0.03; Hedge's *g* = 0.55) and stress (*p* = 0.003; Hedge's *g* = 0.70). However, the increase was non-significant between baseline and follow-up. No main effects were found on overall mindfulness, its other facets, and the CERQ subscales. The results of the Bayesian analyses revealed similar findings to the inferential statistics and can be found in the Supplementary Material.

## Discussion

### Mindfulness

There has been evidence of increased mindfulness through mindfulness-based interventions in prison and psychiatric populations (8, 28). For forensic inpatients, prior research has demonstrated an increasing trend in the *observing* facet in their study on mindful-yoga training with forensic inpatients (34). This facet examines one's sensory awareness and has been argued to develop earlier in the process of learning mindfulness. Likewise, An et al. (44) found significant increases in the *observing* and *non-reactivity* facets in prisoners who completed a mindfulness training as compared to a waitlist control. In contrast, we found a significant increase in the *describing* facet which was driven by the change between baseline and post-intervention. The *describing* facet explores an individual's ability to label and share their experiences using words, and it negatively correlates with alexithymia [i.e., difficulty in describing one's emotional states; (35)]. Day (45) summarized research that suggests offenders score highly on alexithymia. Furthermore, treatment engagement is arguably affected by their ability to describe feelings. Thus, changes in the *describing* facet may be important for this population and may provide a potential understanding for how the MTP is helping participants. In addition, learning to describe one's emotional state is a skill that can potentially benefit other treatments. For example, many participants were engaged in therapy and other groups for which expressing one's feelings would be pertinent. Exploring this impact is beyond the scope of our study, but future researchers could elucidate other ways mindful-yoga impacts patient well-being and treatment.

### Perceived Stress

Researchers of incarcerated populations have noted prison is a stressful environment (2), and the present investigation found a significant decrease in perceived stress from baseline to post-intervention, but not at follow-up. While quantitative and qualitative studies examining the impact of mindfulness and/or yoga on stress have shown significant reductions in prison populations [e.g., (28)] and in psychiatric inpatients [e.g., (46)], others have not found reductions in perceived stress [e.g., (34)]. Interestingly, although Sistig et al. (34) did not have significant quantitative findings, the forensic inpatients qualitatively reported mindful-yoga training aided in stress management. It is possible the equivocal findings are due to external factors. The stressful environment and circumstantial factors (e.g., changes in medication) could impact participant responses. Qualitative reports may offer a more comprehensive understanding of how participants perceive stress; highlighting the importance of such feedback in this line of research.

### Cognitive and Emotion Regulation Strategies

Howells et al. (14) highlighted how forensic inpatients may have poor self-regulation strategies and argued how mindfulness training and practice may aid in developing such skills. In exploring the impact of the MTP on the use of different cognitive and emotion regulation strategies, no significant changes were noted. These findings are aligned with another study examining mindfulness training with early psychosis patients (25, 47). It is possible that these nonsignificant findings are due to the variable psychometric properties of the subscales. When focusing on the three subscales with the strongest properties (i.e., positive refocusing, positive reappraisal, and other-blame), benefit-consistent changes are noted. In particular, the increase in positive refocusing persists into follow-up. Positive refocusing is a strategy wherein the individual seeks to focus on pleasant ideas rather than what might be causing distress. As the MTP program emphasizes directing attention in a positive direction and includes in-depth discussions about happiness and what that means for an individual, it is understandable that positive refocusing emerges as a potentially important strategy. Further exploration of these different strategies would be important.

### Long-Term Effects of MTP

For the two significant changes observed in this study, significant pairwise comparisons were noted only between baseline and post-intervention. At the 3-months follow up, the *describe* facet had decreased while stress had increased again albeit the means did not reach baseline levels. In contrast, the qualitative findings on stress reported by Sistig et al. (34) appeared to be maintained at follow-up along with their benefit consistent trend for the *observe* mindfulness facet. It should be noted that our follow-up period (3-months) is slightly longer than their 2-months follow-up. Taken together, the findings suggest the long-term effects of the MTP are unclear and warrant further exploration. In particular, it would be of interest to explore how the participants engage with mindfulness and yoga following program completion. How much participants engage in the taught activities in the subsequent months would be an in important moderating factor. In addition, exploring important life changes between the completion of the program and the follow-up would be of value. For example, changes in social interaction or treatment could impact findings.

### Limitations and Future Directions

There are seven key limitations to be noted: (1) small sample size and large attrition, (2) the increase familywise error rate from multiple statistical analyses was not controlled due to the small sample and preliminary nature of the study, (3) measures are limited by psychometric strength and focus on clinical measures, (4) qualitative measures on program acceptability were not included, (5) program fidelity was not assessed, (6) most patients participated in other therapeutic groups, and (7) statements on the generalizability of our findings are limited by the small sample and the inclusion of only one institution. Based on these limitations, we recommend future studies include more comprehensive quantitative measures (i.e., including nonclinical outcomes): (1) at baseline, post-intervention, and follow-up, and (2) before and after each weekly session. We further suggest adding qualitative measures regarding program feasibility, impact, and the types of mindfulness practiced independently. It would have been of interest to know how patients felt about the program, whether they would recommend it to others, and what benefits they perceived from participation. Finally, a controlled research design with a longer follow-up period is needed to ascertain the impact of such programs.

This is the second study examining the impact of mindful-yoga on forensic inpatients, and the first exploring Canadians. The significant findings may support the MTP's use as an adjunctive intervention; specifically, the reduction in stress and increase in the *describing* facet may support patients with transitioning into the facility and engaging in other treatments. As a vulnerable population who are in need of well-researched adjunctive therapies, the present pilot study is an important attempt at quantifying the effects of mindfulness and yoga training on forensic inpatients and supports the potential for expansion to other facilities in Canada.

## Data Availability Statement

The datasets presented in this article are not readily available because our ethics certificate did not include sharing any data with a third party even if it is non-identifiable. Requests to access the datasets should be directed to bassam.el-khoury@mcgill.ca.

## Ethics Statement

The studies involving human participants were reviewed and approved by the ethics review board of l'Institut Philippe-Pinel de Montréal and McGill University. The patients/participants provided their written informed consent to participate in this study.

## Informed Consent

Written informed consent was obtained from all individual participants included in the study.

## Author Contributions

CS, BK, and EP-G conducted the literature searches and designed the study. EP-G aided in the study design, collected data, and contributed to editing the final manuscript. MP, MF, VM, and AW aided in analyzing the data and creating the tables as well as the writing and editing of the manuscript. BK supported the study design and provided feedback on the final manuscript. All authors have approved the final version of the manuscript for submission.

## Conflict of Interest

The authors declare that the research was conducted in the absence of any commercial or financial relationships that could be construed as a potential conflict of interest.
