# Supplementary material for: Evaluating the Use of Mindfulness and Yoga Training on Forensic Inpatients: A Pilot Study
Source: Front Psychiatry. 2020 Dec 10;11:614409. doi: 10.3389/fpsyt.2020.614409 (PMC7758348; doi:10.3389/fpsyt.2020.614409)
Supplement: Supplementary file 1 [file Data_Sheet_1.docx]

**Supplementary Material**

**Intervention effects on measured outcomes: Bayesian analysis**

To further assess our findings, a Bayesian repeated measures approach was used with uninformative prior odds (due to the scarcity and limitations of previous studies). The null hypothesis is that there is no effect of time on outcome, while the alternative hypothesis posits there is an effect. To classify the Bayes factor and the relative evidence supporting a hypothesis, descriptive labels are used (Goss-Sampson, 2020; Jeffreys, 1961). These range from anecdotal to moderate, to strong, to very strong, and to decisive. The results are summarized in Table 1 and 2.

All analyses had error percentages are under 10% which is considered negligible (Goss-Sampson, 2020). Prior probabilities were set such that each model is equal (i.e., .5 for both the null and alternative hypotheses). For the *describing* facet, the Bayes factor indicated the model with time as a predictor is 2.45 times more likely than the null model. This suggested anecdotal evidence for the alternative hypothesis. Post-hoc comparisons of baseline and post-training, and baseline and follow-up revealed posterior odds of 3.81 and 1.11 against the null hypothesis. This indicated moderate and anecdotal evidence supporting the alternative hypothesis respectively. For stress, the Bayes factor indicated the model with time as a predictor is 14.21 times more likely than the null model. This suggested strong evidence for the alternative hypothesis. Post-hoc comparisons of baseline and post-training revealed posterior odds of 22.89 against the null hypothesis; indicating strong evidence in favor of the alternative hypothesis. However, when comparing baseline with follow-up, posterior odds revealed anecdotal evidence favoring the null hypothesis. All other outcomes had either anecdotal or moderate support for the null hypothesis.

**Table 1:** *Statistics of Bayesian repeated measures ANOVA tests for the total score on mindfulness (and its five facets), perceived stress, and cognitive emotion regulation strategies. Prior and posterior probabilities, Bayes factors, error percentages, and descriptive labels are listed.*

| **FFMQ** | ***P(M)*** | ***P(M\|data)*** | ***BF_10_*** | ***Error %*** | ***Descriptive Label*** |
| --- | --- | --- | --- | --- | --- |
| Total Score | 0.50 | 0.29 | 0.41 | 0.70 | Anecdotal |
| Acting with awareness | 0.50 | 0.23 | 0.30 | 0.69 | Moderate |
| Describing | 0.50 | 0.71 | 2.45 | 1.15 | Anecdotal* |
| Non-judging | 0.50 | 0.20 | 0.24 | 0.85 | Moderate |
| Non-reactivity | 0.50 | 0.32 | 0.48 | 0.62 | Anecdotal |
| Observing | 0.50 | 0.29 | 0.42 | 0.96 | Anecdotal |
| **CERQ** | ***P(M)*** | ***P(M\|data)*** | ***BF_10_*** | ***Error %*** | ***Descriptive Label*** |
| Acceptance | 0.50 | 0.17 | 0.21 | 1.04 | Moderate |
| Catastrophizing | 0.50 | 0.28 | 0.39 | 0.77 | Anecdotal |
| Other-blame | 0.50 | 0.16 | 0.19 | 0.76 | Moderate |
| Positive re-appraisal | 0.50 | 0.20 | 0.25 | 0.84 | Moderate |
| Positive refocusing | 0.50 | 0.46 | 0.86 | 0.61 | Anecdotal |
| Putting into perspective | 0.50 | 0.39 | 0.65 | 0.81 | Anecdotal |
| Refocus on planning | 0.50 | 0.34 | 0.51 | 0.73 | Anecdotal |
| Rumination | 0.50 | 0.16 | 0.19 | 0.64 | Moderate |
| **PSS** | ***P(M)*** | ***P(M\|data)*** | ***BF_10_*** | ***Error %*** | ***Descriptive Label*** |
| Perceived Stress | .50 | .93 | 14.21 | .54 | Strong* |

*Note. **In favor of the alternative hypothesis. BF_10_, Bayes Factor; CERQ, Cognitive Emotion Regulation Questionnaire; Error %, Error Percentage; FFMQ, Five-facet Mindfulness Questionnaire; P(M), Prior Probabilities; P(M|data); Posterior Probabilities; PSS, Perceived Stress Scale.

**Table 2:** *Post-hoc comparisons at baseline, post-intervention and follow-up for describing facet and perceived stress. Prior odds, posterior odds, Bayes factors, error percentages, and descriptive labels are listed.*

| **Describing Facet** | ***Prior Odds*** | ***Posterior Odds*** | ***BF_10,U_*** | ***Error %*** | ***Descriptive Label*** |
| --- | --- | --- | --- | --- | --- |
| Baseline to Post-Intervention | 0.59 | 3.81 | 6.48 | <.001 | Moderate* |
| Baseline to Follow-up | 0.59 | 1.11 | 1.88 | <.001 | Anecdotal* |
| **Perceived Stress** | ***Prior Odds*** | ***Posterior Odds*** | ***BF_10,U_*** | ***Error %*** | ***Descriptive Label*** |
| Baseline to Post-Intervention | 0.59 | 22.89 | 38.97 | <.001 | Strong* |
| Baseline to Follow-up | 0.59 | 0.50 | 0.85 | .004 | Anecdotal |

*Note. **In favor of the alternative hypothesis. BF_10,U_, Uncorrected Bayes Factor; Error %, Error Percentage.

REFERENCES

Jeffreys H. *Theory of probability (3rd Ed.).* Oxford: Oxford University Press (1961).

Goss-Sampson M. *Bayesian Inference in JASP: A Guide for Students* (2020). doi: 10.17605/OSF.IO/CKNXM
